# Dynamic Contrast-Enhanced MRI to Study Atherosclerotic Plaque Microvasculature

**DOI:** 10.1007/s11883-016-0583-4

**Published:** 2016-04-26

**Authors:** Raf H. M. van Hoof, Sylvia Heeneman, Joachim E. Wildberger, M. Eline Kooi

**Affiliations:** Department of Radiology, Maastricht University Medical Center (MUMC), P.O. Box 5800, 6202 AZ Maastricht, The Netherlands; CARIM School for Cardiovascular Diseases, Maastricht University, P.O. Box 616, Maastricht, 6200 MD The Netherlands; Department of Pathology, Maastricht University Medical Center (MUMC), P.O. Box 5800, Maastricht, 6202 AZ The Netherlands

**Keywords:** Atherosclerosis, Dynamic contrast-enhanced MRI, Microvasculature, Quantification

## Abstract

Rupture of a vulnerable atherosclerotic plaque of the carotid artery is an important underlying cause of clinical ischemic events, such as stroke. Abundant microvasculature has been identified as an important aspect contributing to plaque vulnerability. Plaque microvasculature can be studied non-invasively with dynamic contrast-enhanced (DCE-)MRI in animals and patients. In recent years, several DCE-MRI studies have been published evaluating the association between microvasculature and other key features of plaque vulnerability (e.g., inflammation and intraplaque hemorrhage), as well as the effects of novel therapeutic interventions. The present paper reviews this literature, focusing on DCE-MRI methods of acquisition and analysis of atherosclerotic plaques, the current state and future potential of DCE-MRI in the evaluation of plaque microvasculature in clinical and preclinical settings.

## Introduction

Rupture of a vulnerable atherosclerotic plaque is an important underlying cause of clinical ischemic events, such as stroke [1]. Therefore, visualization of vulnerable plaques may aid in the identification of patients who have an increased risk for a clinical event. Inflammatory cells play an important role during the development and progression of atherosclerosis [2]. Within atherosclerotic plaques, activated macrophages have a high metabolic rate, inducing hypoxia which stimulates the formation of new microvessels originating from the outer layer of the vessel wall, the adventitia [3, 4]. These newly formed microvessels generally have impaired endothelial integrity, which can lead to extravasation of inflammatory cells and erythrocytes from the microvessel lumen into plaque tissue [4]. Extravasation of erythrocytes is generally considered as an important contributing factor to intraplaque hemorrhage (IPH) [5]. Because lipids constitute 40 % of the erythrocyte membrane [5], extravasation of erythrocytes leads to increased cholesterol deposition in the plaque tissue, which in turn stimulates further recruitment of inflammatory cells. All the above biological events, especially leaky plaque microvasculature, are considered key features in plaque destabilization [6].

The microvasculature in plaques are very small (up to ∼100 μm in diameter) but can be studied non-invasively by several imaging modalities, including contrast-enhanced ultrasound (CEUS) [7, 8], positron emission tomography (PET) [9–11], and dynamic contrast-enhanced magnetic resonance imaging (DCE-MRI) [12]. MRI is a well-established imaging modality that can be used to visualize the main plaque components: areas of IPH, the lipid-rich necrotic core, and fibrous cap status [13–15]. Early studies developed MRI for the detection of morphological and chemical components by studying specimen from surgery (carotid endarterectomy (CEA)). These ex vivo specimens were advantageous for testing and developing MRI sequences, but the lack of blood precludes studying of the dynamics from DCE [16, 17].

In recent years, a number of studies have applied DCE-MRI to study atherosclerotic plaque microvasculature. The present paper reviews the current state and future potential of DCE-MRI in the evaluation of plaque microvasculature with applications in animals and patients. First, because the methods of DCE-MRI are now well-developed and widely applied but are not familiar to a general audience, we begin with principles and acquisition methods of DCE-MRI and methods for (semi)quantitative analysis of DCE-MRI data. Second, an overview is given of publications on DCE-MRI of plaque microvasculature (Table 1) used to study one of the following aspects: (1) associations between plaque microvasculature and other plaque features, (2) longitudinal changes in plaque microvasculature, (3) comparison of different animal groups and human subjects with a different cardiovascular risk profile, and (4) evaluation of therapy response. Finally, future challenges and potential for DCE-MRI to study plaque microvasculature will be discussed.

**Table 1:** Overview of DCE-MRI studies of atherosclerotic plaque microvasculature. Overview of studies investigating the atherosclerotic plaque microvasculature using dynamic contrast-enhanced MRI: subjects (human or rabbits), analysis method (quantitative or semi-quantitative), main study purpose, and study outcome are shown

Reference	Subjects	Main study purpose	Main study outcome
Chen et al. [18]	Patients with CVD (AIM-HIGH Trial [19]	Scan-rescan reproducibility	Moderate reproducibility for K^trans^ (Patlak) with a 25 % coefficient of variation. To limit dropout, intensive operator training, optimized imaging, and quality control is required
Kerwin et al. [20]	CEA patients	Method development	Development of a motion correcting and noise reducing algorithm for the analysis of DCE-MRI of carotid arteries
Kerwin et al. [21]	Patients with a carotid lesion ≥ AHA type IV	Method comparison	Quantitative enhancement characteristics, such as K^trans^ (Patlak), depend on the used contrast medium (gadobenate dimeglumine vs gadodiamide)
Ramachandran et al. [22]	Humans with CVD risk	Method development	Development of a registration method for alignment of different time frames of DCE-MRI of carotid arteries
Chen et al. [23]	Humans with advanced carotid disease	Method development	Extended graphical model exhibits a reduced bias in K^trans^ estimation compared to the Patlak model
Van Hoof et al. [24]	Symptomatic patients (30–99 % carotid stenosis)	Method comparison	Comparison between phase- and magnitude-based vascular input functions and resulting effect on pharmacokinetic parameters. No signal saturation due to blood flow for phase-based determined vascular input function
Calcagno et al. [25]	Humans with CVD risk	Method development	Demonstration of feasibility of simultaneous VIF and vessel wall imaging (extended Tofts)
Wan et al [26]	NZW Rabbit^a,b^	Method development	Spatio-temporal texture based features (like AUC) are able to distinguish between vulnerable and stable plaques.
Calcagno et al. [27]	NZW Rabbit^c^	Method comparison	Excellent reproducibility of DCE-MRI derived AUC (interscan, intraobserver, and interobserver ICCs > 0.75, *P* < 0.001)
Wu et al. [28••]	NZW Rabbit1	Method development	Demonstration of feasibility of simultaneous VIF and vessel wall imaging with accurate estimation of pharmacokinetic parameters (Patlak)
Calcagno et al. [29]	NZW Rabbit3	Histological validation	Positive correlation (*ρ* = 0.89, *p* = 0.016) between AUC and amount of neovessels in the intima
Calcagno et al. [30•]	NZW Rabbit3	Histological validation	3D DCE-MRI (AUC (*ρ* = 0.45) and K^trans^ (Patlak) (*ρ* = 0.38)) is able to quantify microvascular permeability in the entire abdominal aorta plaque
Chen et al. [31]	NZW Rabbit^d^	To study plaque progression	DCE-MRI (AUC) is able to quantitatively assess temporal changes of atherosclerotic plaques over a period of 3 months
Kim et al. [32]	NZW Rabbit3	Validation of a chip for the development of nanomedicines	Increased AUC for atherosclerotic animals compared to control animals. Lipid-polymer hybrid nanoparticle translocation is correlated with AUC (*ρ* = 0.79, *p* < 0.0001)
Lobatto et al. [33]	NZW Rabbit3	Evaluation of glucocorticoid treatment for atherosclerosis	DCE-MRI (AUC) reveals early changes in plaque microvascular permeability after liposomal glucocorticoid treatment
Vucic et al. [34]	NZW Rabbit3	Evaluation of pioglitazone treatment for atherosclerosis	DCE-MRI (AUC) can demonstrate the anti-inflammatory effect of pioglizatone on atherosclerotic plaques
Vucic et al. [35]	NZW Rabbit3	Evaluation of LXR agonist R211945 treatment for atherosclerosis	DCE-MRI (AUC) showed a trend towards a decreased microvasculature after treatment with atorvastatin
Chen et al. [36]	Patients with >50 % carotid stenosis	Comparison of plaque Components	K^trans^ and v_p_ (Patlak) differed significantly between plaque components (lipid core, IPH, calcifications, loose matrix, and fibrous tissue), except between calcifications and IPH.
Calcagno et al. [37]	Patients with CHD or CHD risk equivalent	Correlation with ^18^F-FDG PET-CT	Weak, inverse relationship between inflammation (^18^F-FDG PET-CT, mean TBR) and plaque perfusion (DCE-MRI, K^trans^ (extended TK))
Dong et al. [38]	Humans (carotid plaque thickness ≥2 mm)	Evaluation of intensive lipid therapy in the treatment of atherosclerosis	Intensive lipid therapy (using atorvastatin, niacin, and colesevelam) results in a reduction in K^trans^ (Patlak) after one year
Gaens et al. [39]	Symptomatic patients (30–99 % carotid stenosis)	Pharmacokinetic model comparison	The Patlak model is the most suited quantitative model for description of carotid plaque microvasculature
Kerwin et al. [40]	CEA Patients	Validation against microvasculature on histology	Strong correlation (*ρ* = 0.80, *p* < 0.001) between DCE-MRI and histological measured fractional vascular areas
Kerwin et al. [41]	CEA Patients	Validation against microvasculature and inflammation on histology	K^trans^ (Patlak) is a quantitative and non-invasive marker of plaque inflammation (*ρ* = 0.75, *p* < 0.001) and microvasculature (*ρ* = 0.71, *p* < 0.001)
Kerwin et al. [42]	CEA Patients	Validation against microvasculature and inflammation on histology	Adventitial K^trans^ (Patlak) was significantly correlated with the amount of microvasculature (*ρ* = 0.41, *p* = 0.04) and macrophages (*ρ* = 0.49, *p* = 0.01)
Mani et al. [43]	Humans with and without exposure to particle matter	Risk stratification	High exposure to particle matter may be associated with plaque neovascularization, measured with DCE-MRI (AUC)
O’Brien et al. [44••]	Patients with CVD (AIM-HIGH Trial) [19]	Association of DCE-MRI with statin therapy	Shorter duration of statin therapy before occurrence of clinical event is associated with increased v_p_ (Patlak)
Sun et al. [45•]	Symptomatic patients (ischemic event <6 m)	Correlation between DCE-MRI (K^trans^) and presence of IPH	Presence of IPH was associated with an increase of 28 % of adventitial K^trans^ (Patlak)
Truijman et al. [46]	Symptomatic patients (30–69 % carotid stenosis)	Correlation with ^18^F-FDG PET-CT	Weak, positive relationship between inflammation (^18^F-FDG PET-CT, TBR) and plaque perfusion (DCE-MRI, K^trans^ (Patlak))
Wang et al. [47•]	Human (carotid plaque thickness ≥2 mm)	Correlation with ^18^F-FDG PET-CT	Correlation between ^18^F-FDG PET (TBR) and DCE-MRI (K^trans^, Patlak) measurements varied with clinical conditions (symptomatic status)

*DCE-MRI* dynamic contrast-enhanced MRI, ^*18*^ *F-FDG* ^18^fluorine-fluorodeoxyglucose, *PET*-*CT* positron emission tomography/computed tomography, *AUC* area under the curve, *NIRF* near-infrared fluorescence, *CVD* cardiovascular disease, *CEA* carotid endarterectomy, *CHD* coronary heart disease, *TBR* target-to-background ratio, *NZW* New Zealand White

^a^Atherosclerosis was induced by a balloon injury of the aorta in combination with a high cholesterol-enriched diet (1.0 %)

^b^Pharmacologic triggering was performed to stimulate plaque disruption

^c^Atherosclerosis was induced by a balloon injury of the aorta in combination with a low cholesterol enriched diet (<1.0 %) combined with palm oil

^d^Atherosclerosis was induced by a balloon injury of the aorta in combination with a low cholesterol enriched diet (<1.0 %)

## DCE-MRI Methods to Study Plaque Microvasculature

### Principles of DCE-MRI

**Fig. 1:**
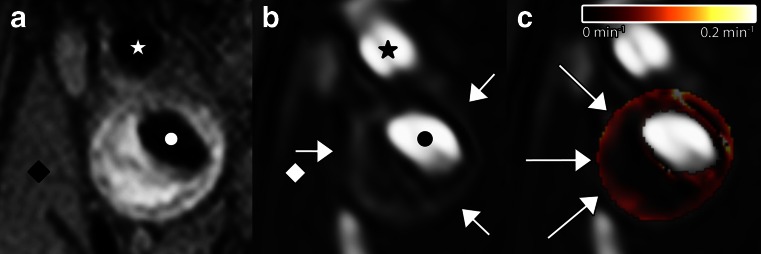
MR images (**a**–**c**) of a transverse section of the carotid plaque in the internal carotid artery from a 64-year-old man. In **a**, A *black* blood T1-weighted turbo spin echo MR image as an anatomical reference. In this image, the vessel lumen (*circle*) appears in *black*. The atherosclerotic plaque of this patient appears hyperintense compared to the sternocleidoid muscle (*diamond*). In **b**, a three-dimensional T1-weighted fast field-echo dynamic contrast-enhanced MR image that is acquired 6 min after contrast injection is shown. In this image, the vessel lumen (*circle*) appears bright compared to the atherosclerotic plaque and surrounding tissues. A ring of enhancement can be observed at the outer part of the vessel wall (indicated by *white arrows*), which is attributed to the microvasculature originating from the adventitia. Finally, in **c**, a parametric K^trans^ map is overlaid on DCE-MRI image shown in **b**. In this parametric map, voxel wise determined K^trans^ values are color encoded from 0 to 0.2 min^−1^. Within this overlay, the lipid-rich necrotic core in the center of the plaque, exhibits low K^trans^ values (*dark*), while the highly vascularized adventitia (high K^trans^ values) at the outer rim (indicated by the *arrows*) is clearly visualized (*red regions*). *Circle*, internal carotid artery; *star*, external carotid artery; *diamond*, sternocleidoid muscle. Figure adapted from Truijman et al. [46]

All DCE-MRI experiments require serial acquisition of MR images acquired in a brief time interval to study atherosclerotic plaque microvasculature (Fig. 1). After acquisition of anatomical references (Fig. 1a), the first images of the series, acquired before contrast injection, are used to determine baseline signal intensity of the atherosclerotic plaque tissue. Bolus injection of a low molecular weight non-specific Gadolinium-based contrast medium follows, and image acquisition is continued for several minutes. During this period, the bolus of contrast medium will be distributed, resulting in signal enhancement of the blood vessel lumen, vessel wall due to leakage of the contrast medium through damaged endothelial, and other tissues, such as skeletal muscle (Fig. 1b). In this image, the vessel lumen (circle) appears bright. A ring of enhancement in the outer (adventitial layer) part of the vessel wall (indicated by white arrows) can be clearly observed. The signal enhancement in the vessel wall depends on flow, microvascular density, the ability of the contrast medium to leak from the microvasculature into the extravascular extracellular space, and reflux. After analysis of the DCE-MR images, parametric maps (Fig. 1c) of the resulting parameter can be generated, indicating local leaky plaque microvasculature. In DCE-MRI studies of atherosclerosis to date, linear or cyclic Gadolinium-based contrast media have been used.

DCE-MRI studies of brain and tumor perfusion mostly use a contrast medium injection rate of 2 ml/s (typically 0.1 mmol/kg). Such fast injection rates result in quick passage of the bolus through the vessel and a high-contrast medium peak concentration, necessitating a higher temporal resolution for MR acquisition and compromising spatial resolution. For the evaluation of carotid atherosclerosis using DCE-MRI, however, high spatial resolution is required for accurate visualization of the vessel wall. Therefore, some DCE-MRI studies [24, 39] have used a slower injection rate of 0.5 ml/s in plaque imaging. Previous research has shown that a high injection rate is most beneficial for high K^trans^ values (>0.2 min^−1^) [48]. Typically, within the atherosclerotic lesion, mean K^trans^ values below 0.15 are reported [24], and therefore, a lower injection rate may be applied.

Signal enhancement-time curves of DCE-MR images can be analyzed voxelwise or using a region-of-interest. Especially in the voxelwise analysis, movement of the subject during acquisition of the different DCE-MRI sequence time frames may pose a problem. A solution is to manually shift individual time frames to correctly align the images, or alternatively, to use post-processing methods for automated movement correction and noise reduction [20, 22].

### Pulse Sequences for DCE-MRI of Plaque Microvasculature

Currently, two main categories of pulse sequences for DCE-MRI of atherosclerotic plaque microvasculature are employed: “bright blood” or “black blood”. Black blood imaging facilitates improved delineation of the inner vessel wall, whereas bright blood imaging enables to determine the CM concentration in the vessel lumen for each patient individually. Because the luminal CM concentration cannot be quantified accurately, quantitative analysis of black blood DCE-MRI with pharmacokinetic models can only be performed using a reference region model [49] or previously determined generalized input functions [50]. Recently, dedicated imaging methods have been proposed combining bright and black blood images in an interleaved fashion, allowing improved delineation of the vessel wall from black blood images as well as extraction of vascular input function based on lumen signal intensity from bright blood images [25, 28••].

A compromise between the desired spatial and the required temporal resolution must be made regardless of the imaging method used. Current studies (both in rabbits and patients) employed an in-plane spatial acquisition resolution of approximately 0.5 × 0.5 mm^2^. The preclinical rabbit studies have employed a temporal resolution of 5 s for 2D acquisition techniques and lower temporal resolution (30 s) for 3D techniques. In patient studies, the temporal resolution ranges from 15 to 30 s per time frame.

### Semi-Quantitative Assessment of the Microvasculature

The microvasculature can be assessed semi-quantitatively using the area-under-the-curve (AUC) of the (relative) signal enhancement curve. This requires that start- and end-time points are selected over which the AUC will be calculated. Generally, the moment of contrast arrival in the tissue of interest is chosen as the starting point, and the end time point is chosen empirically. It must be noted that when the end point is chosen relatively close to the contrast injection, the AUC reflects early contrast arrival, whereas a later end point will cause the AUC to reflect total leakage (and entrapment) of contrast medium in the plaque tissue.

The main advantage of semi-quantitative analyses is the relatively easy implementation. However, the information is limited because there is no direct relationship between the AUC and (patho)physiological parameters. Although research in the field of oncology [51] has shown that the AUC reflects pathophysiology, it does so non-specifically, meaning that one particular AUC value can indicate a number of biological properties. Thus, an increased AUC can indicate increased leakage of the contrast medium from the microvasculature, increased density of microvessels, increased flow through the microvasculature, a decrease in reflux from the extracellular extravascular space to the microvasculature, or a combination of these. Therefore, changes or differences in the AUC may result from a variety of phenomena so that it may be difficult to attribute these changes to a single, underlying physiological cause. Similarly, effects of therapeutic interventions may potentially be obscured using the AUC. Another drawback of semi-quantitative analysis is the difficulty of direct comparison of results between studies because the AUC also depend on settings of the MR system, such as receiver gain.

#### Validation of Semi-Quantitative DCE-MRI Parameters

Validation of semi-quantitative DCE-MRI was performed in several balloon injured cholesterol-fed New Zealand White rabbit studies. It was found that the AUC positively correlated with microvessel count in the intima of histological specimens (Pearson’s *ρ* of 0.89 (*p* = 0.016) and 0.91 (*p* = 0.011) for the AUC 2 and 7 min after contrast injection, respectively) [29]. Furthermore, later research [27] showed a good interscan and excellent intra- and inter-observer reproducibility (all ICCs > 0.75, *p* < 0.01).

Another atherosclerotic rabbit study compared two three-dimensional (3D) high spatial resolution DCE-MRI sequences (3D turbo field echo (TFE) with motion-sensitized-driven equilibrium (MSDE) preparation and a 3D turbo spin echo (TSE) sequence) [30•]. A moderate Pearson correlation was found between AUC and ex vivo permeability measurements using Evans Blue (an albumin-binding dye used for quantification of ex vivo vascular permeability) near-infrared fluorescence (NIRF) (*ρ* = 0.45 for 3D TFE MRI and *ρ* = 0.39 for 3D TSE MRI). In addition, a fourfold improvement of temporal resolution was achieved when using compressed sensing by retrospective undersampling and reconstruction. In another study, comparison between in vivo (3D DCE-MRI) and ex vivo (Cy7-labeled near-infrared fluorescence [NIRF]) measures of microvascular permeability in the aortic wall of atherosclerotic rabbits showed a high degree of correlation between both imaging modalities (*r*^2^ = 0.65, *p* < 0.0001) [32].

These studies [27, 29, 30•, 32] have demonstrated reproducible representation of plaque microvasculature through semi-quantitative DCE-MRI parameters.

### Quantitative Assessment of the Microvasculature

#### Pharmacokinetic Modeling

Pharmacokinetic modeling allows the quantification of contrast medium distribution over a tissue of interest with the main advantage of deriving parameters of the in vivo physical quantities of the amount, flow, and leakiness of the microvasculature.

**Fig. 2:**
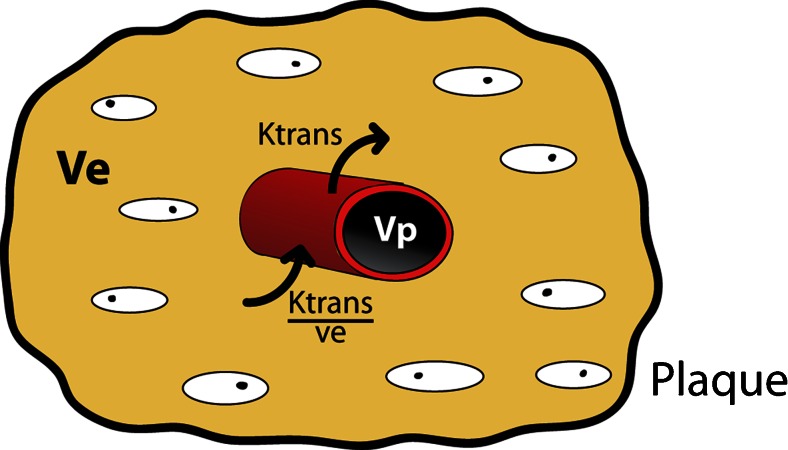
Schematic representation of parameters used in pharmacokinetic models for analysis of atherosclerotic plaque microvasculature. Within a single region of interest or voxel, the fractional blood volume (microvasculature) is represented by v_p_, while the fraction of the extracellular extravascular space is represented by v_e_. Contrast medium transfer rate from the microvasculature to the extracellular extravascular space is given by K^trans;^ the reflux is described by K^trans^/v_e_. In most DCE-MRI studies, an extracellular contrast medium with a low molecular weight is used. For quantitative data analysis, therefore, a two-compartment model can be used (i.e., vascular and extracellular extravascular compartments). Based on this general concept and setting various assumptions, several different quantitative models can be derived. An overview of these models is presented in Table 2

A number of quantitative DCE-MRI data analysis models have been applied in the evaluation of atherosclerotic plaque microvasculature (Table 2). These models describe the relationship between the concentration of the (extracellular) contrast medium in the blood plasma (C_p_) and the extracellular extravascular space (C_e_) according to the two-compartment model and using the parameters K^trans^, v_e_, and v_p_. K^trans^, the transfer constant of contrast medium from plasma to the tissue compartment, serves as an indicator of blood supply and vessel permeability within the atherosclerotic tissue. The parameters v_e_ and v_p_ represent the extravascular extracellular space and the plasma fractional volume, respectively. A schematic representation of the physiological meaning of the parameters is shown in Fig. 2.

**Table 2:** Overview of quantitative DCE-MRI models used in the analysis of atherosclerosis. Quantitative pharmacokinetic models used for the analysis of atherosclerosis based on the two-compartment model. The modified/extended Tofts and Kermode model is the analytical solution for the two-compartment model. The extended graphical model is based on a second order Taylor expansion of the modified/extended Tofts and Kermode model

	Mathematical description	Parameters		
Two-compartment model	$$ \begin{array}{l}\frac{{\mathrm{dC}}_{\mathrm{e}}\left(\mathrm{t}\right)}{\mathrm{dt}}=\frac{{\mathrm{K}}^{\mathrm{t}\mathrm{rans}}}{{\mathrm{V}}_{\mathrm{e}}}\left({\mathrm{C}}_{\mathrm{p}}\left(\mathrm{t}\right)-{\mathrm{C}}_{\mathrm{e}}\left(\mathrm{t}\right)\right)\hfill \\ {}{\mathrm{C}}_{\mathrm{t}}\left(\mathrm{t}\right)={\mathrm{v}}_{\mathrm{p}}{\mathrm{C}}_{\mathrm{p}}\left(\mathrm{t}\right)+{\mathrm{v}}_{\mathrm{e}}{\mathrm{C}}_{\mathrm{e}}\left(\mathrm{t}\right)\hfill \end{array} $$			
	Mathematical description	K^trans^	v_e_	v_p_
Modified/extended Tofts and Kermode (TK)	${\mathrm{C}}_{\mathrm{t}}\left(\mathrm{t}\right)={\mathrm{v}}_{\mathrm{p}}{\mathrm{C}}_{\mathrm{p}}\left(\mathrm{t}\right)+{\mathrm{K}}^{\mathrm{t}\mathrm{rans}}{\displaystyle \underset{0}{\overset{\mathrm{t}}{\int }}{\mathrm{C}}_{\mathrm{p}}\left(\mathrm{t}\right){\mathrm{e}}^{-\frac{{\mathrm{K}}^{\mathrm{t}\mathrm{rans}}}{{\mathrm{v}}_{\mathrm{e}}}\left(\mathrm{t}-\uptau \right)}\mathrm{d}\uptau}$	X	X	X
Tofts and Kermode	${\mathrm{C}}_{\mathrm{t}}\left(\mathrm{t}\right)={\mathrm{K}}^{\mathrm{t}\mathrm{rans}}{\displaystyle \underset{0}{\overset{\mathrm{t}}{\int }}{\mathrm{C}}_{\mathrm{p}}\left(\mathrm{t}\right){\mathrm{e}}^{-\frac{{\mathrm{K}}^{\mathrm{t}\mathrm{rans}}}{{\mathrm{v}}_{\mathrm{e}}}\left(\mathrm{t}-\uptau \right)}\mathrm{d}\uptau}$	X	X	
Patlak	${\mathrm{C}}_{\mathrm{t}}\left(\mathrm{t}\right)={\mathrm{v}}_{\mathrm{p}}{\mathrm{C}}_{\mathrm{p}}\left(\mathrm{t}\right)+{\mathrm{K}}^{\mathrm{t}\mathrm{rans}}{\displaystyle \underset{0}{\overset{\mathrm{t}}{\int }}{\mathrm{C}}_{\mathrm{p}}\left(\mathrm{t}\right)\mathrm{d}\uptau}$	X		X
Extended Graphical Model	${\mathrm{C}}_{\mathrm{t}}\left(\mathrm{t}\right)={\mathrm{v}}_{\mathrm{p}}{\mathrm{C}}_{\mathrm{p}}\left(\mathrm{t}\right)+{\mathrm{K}}^{\mathrm{t}\mathrm{rans}}{\displaystyle \underset{0}{\overset{\mathrm{t}}{\int }}{\mathrm{C}}_{\mathrm{p}}\left(\mathrm{t}\right)\mathrm{d}\uptau -\frac{{\mathrm{K}}^{{\mathrm{t}\mathrm{rans}}^2}}{{\mathrm{v}}_{\mathrm{e}}}}{\displaystyle \underset{0}{\overset{\mathrm{t}}{\int }}{\displaystyle \underset{0}{\overset{\uptau_1}{\int }}{\mathrm{C}}_{\mathrm{p}}\left({\uptau}_2\right){\mathrm{d}\uptau}_2{\mathrm{d}\uptau}_1}}$	X	X	X

The modified/extended Tofts and Kermode (TK) model [52, 53] is a commonly employed analytical solution for the two-compartment model [54, 55], estimating all three pharmacokinetic parameters (K^trans^, v_e_, and v_p_). The original TK model, which was proposed for the study of multiple sclerosis [56], does not take vascular contribution into account (i.e., v_p_ is assumed to be negligible). The Patlak model [57] assumes that reflux, i.e., transfer of contrast medium from the tissue compartment back to the blood plasma (K^trans^/v_e_), is negligible. Recently, an approximation of the modified TK model has been introduced as an intermediate solution between the modified TK and the Patlak model: the extended graphical model [23]. This model uses the first-order term of a Taylor series from the modified TK model to estimate v_e_.

#### Vascular Input Function

One essential requirement for quantitative analysis of DCE-MRI data is knowledge of the contrast medium concentration in the blood vessel over time, commonly, referred to as the arterial or vascular input function (AIF/VIF). Two main features of the VIF are a high relative peak concentration and a short bolus passage compared to other tissues. Accurate determination of the VIF requires a relatively high temporal resolution, which usually results in compromise with regard to the spatial resolution that can be achieved.

Two strategies can be employed for the determination of VIF. The first strategy is based on the assumption that VIF is similar in all subjects and a generalized population-averaged VIF, obtained from literature or determined in a cohort is used [24, 39, 46, 50]. An advantage of this method is that data acquisition and analysis requirements are simplified [49]. The second strategy involves measurement of patient-specific function, giving the potential advantage of accounting for variations between subjects [58]. Previous research in oncology found comparable results using either method, and the use of population-averaged vascular input functions resulted in increased [50] or comparable [59] reproducibility. In clinical studies of atherosclerotic plaque microvasculature, a generalized VIF is the most commonly chosen method, probably because of the required spatial resolution for accurate imaging of atherosclerotic plaque in the carotid artery. The generalized VIF can be obtained from a separate study cohort where acquisition is performed with a higher temporal resolution and a lower spatial resolution.

The VIF with MRI can be calculated by two different methods. The first method uses the magnitude of the acquired MR signal and is based on conversion of the relative signal enhancement to contrast medium concentration using the Ernst equation [60]. For this conversion, blood relaxation and contrast medium relaxivity rates are taken into account. A second method based on MR signal phase has been developed more recently [61, 62]. First used in brain perfusion studies with dynamic susceptibility MRI [63], the technique is increasingly used in DCE-MRI [24, 64]. Efforts have been made to compare the magnitude- and phase-based techniques [24, 59, 65–67], showing a strong potential for the phase-based technique, allowing accurate VIF quantification. In a DCE-MRI study of 17 symptomatic patients with a mild to severe carotid stenosis, it was found that the magnitude-based VIF resulted in a strong underestimation of lumen contrast medium concentration as compared to the phase-based VIF [24]. Simulations and phantom experiments showed that this underestimation is caused by local blood flow velocity, which leads to saturation of the magnitude MR signal caused by the shortened T_1_ relaxation time in the presence of contrast medium. Analysis of K^trans^ values using population-averaged input functions showed a strong positive correlation between the two methods, although absolute values significantly differed.

#### Validation of Quantitative DCE-MRI Parameters

Histological validation of carotid plaque DCE-MRI has been carried out using reference specimens from patients after carotid endarterectomy (CEA). However, the drawback of all such validation studies is that these are performed in patients scheduled for CEA. Large randomized trials have shown that symptomatic patients with severe ipsilateral stenosis benefit the most from CEA [68]. This population is more likely to have developed advanced atherosclerotic plaques. In addition, the surgeon removes the intima and part of the media of the vessel wall and the adventitia, from which microvasculature originates [6], is missing in the CEA specimen. An additional limitation of the comparison of in vivo MRI with histological measurements as a reference standard is the comparison of a thin histological slice to thicker MR imaging slice (typically 2 mm). Due to the heterogeneous nature of atherosclerotic lesions, this may result in partial volume effects.

Despite these drawbacks, a strong and positive correlation between fractional blood volume derived from in vivo MRI and post-surgical histology (0.80, *p* < 0.001) was found in 16 CEA patients [40]. In addition, a significant Pearson correlation was reported between the transfer constants K^trans^ calculated from in vivo DCE-MRI with postsurgical histologic measurements of the microvessel area (*ρ* = 0.71, *p* < 0.001 for the entire vessel wall K^trans^ and *ρ* = 0.41, *p* < 0.04 for adventitial K^trans^). Additionally, an association between K^trans^ and other postsurgical histological parameters was reported, i.e., macrophage density (*ρ* = 0.75, *p* < 0.001 for the vessel wall K^trans^ and *ρ* = 0.49, *p* < 0.01 for adventitial K^trans^), loose matrix area (*ρ* = 0.50, *p* = 0.01, for vessel wall K^trans^) [41, 42]. It was also shown that K^trans^ and v_p_ differed significantly between different plaque components (lipid core, IPH, calcifications, loose matrix, and fibrous tissue), except between calcifications and IPH [36].

Reproducibility, fit error, parameter uncertainty, and correlation with histology of carotid plaque DCE-MRI were compared for four pharmacokinetic models in patients with mild to severe carotid stenosis [39]. Analysis of 43 patients showed the highest relative fit error for the Tofts model, while the other three models did not differ in this regard. The Patlak model had a significant lower parameter uncertainty for K^trans^ as compared to the other models. Reproducibility was studied in 16 asymptomatic patients with 30–69 % carotid stenosis who underwent imaging twice with several (4.3 ± 2.8) days between the two examinations. Results showed a good reproducibility for all considered pharmacokinetic models (ICC > 0.6, *p* < 0.05) for K^trans^ and significant scan-rescan ICCs for v_e_ (Tofts) and v_p_ (Patlak). Correlation with histologic findings in 13 CEA patients showed significant positive Pearson’s correlation (*ρ* = 0.7; *p* < 0.01) with the entire vessel wall microvasculature for all models, with the exception of the Tofts model. It was concluded that the Patlak model was the most suited of these four models for pharmacokinetic modeling of the microvasculature in atherosclerotic plaques [39]. Another study [23], however, found favorable results for the extended graphical model for simulated and selected in vivo data of carotid plaques with good to excellent image quality. Their results showed that a compromise between noise and bias sensitivity has to be made when choosing between the Patlak and extended graphical models.

The scan-rescan reproducibility of DCE-MRI was also investigated in a multi-center study [18] of 35 subjects with established cardiovascular disease recruited from 15 hospitals. Results showed a moderate reproducibility for K^trans^ with a coefficient of variation of 25 %. The relatively high dropout rate within the study (31.4 %) suggested a need for intensive operator training, an optimized imaging protocol, and quality control.

The dependence of model parameters on contrast medium was investigated in a study comparing two extracellular contrast media [21]. Quantitative analysis of DCE-MR images demonstrated a lower K^trans^ when using gadobenate dimeglumine (0.0846 min^−1^) as compared to gadodiamide (0.101 min^−1^, *p* < 0.01), while no difference in v_p_ was found. In order to facilitate direct comparison of quantitative DCE-MRI parameters between- or in longitudinal studies, the use of the same contrast medium is recommended.

Taken together, despite the recognized limitations, the above studies demonstrate the suitability of quantitative DCE-MRI parameters for reproducibly determining plaque microvasculature characteristics.

## Overview of DCE MRI Studies to Study Plaque Microvasculature

### Association Between DCE-MRI Parameters and Other Plaque Features

Many plaque characteristics and pathological features contribute to the risk for disruption and thrombosis, and studies have been designed to investigate possible associations between plaque microvasculature and other plaque features. In recent years, several studies [31, 37, 45•, 46, 47•] were carried out to investigate associations between DCE-MRI parameters, plaque inflammation, and the presence of IPH. In a preclinical study of cholesterol-fed balloon-injured atherosclerotic rabbits [31], a positive Pearson correlation (*ρ* = 0.70, *p* = 0.01) was found between DCE-MRI derived parameters and histologically determined plaque macrophage content.

The relationship between DCE-MRI parameters and plaque inflammation using ^18^fluorine-fluorodeoxyglucose (^18^F-FDG) PET-computed tomography (CT) has been investigated in several clinical studies [37, 46, 47•]. One study of 49 symptomatic patients with mild to moderate carotid stenosis [46] reported a weak positive correlation (Spearman *ρ* = 0.30, *p* = 0.035) between plaque inflammation (mean Target-to-Background Ratio (TBR) on ^18^F-FDG PET-CT) and plaque perfusion (mean K^trans^). Another study of 33 patients [37] with coronary heart disease (CHD) or CHD risk equivalent and a carotid plaque with TBR ≥ 1.6 on ^18^F-FDG PET-CT [69] found a significant inverse relationship between plaque perfusion (K^trans^) and plaque inflammation on ^18^F-FDG PET-CT of *ρ* = -0.24 (*p* < 0.05). A subsequent study of 41 patients with carotid plaque [47•] found that correlations depend on the clinical condition of patients. Overall, a weak, marginal non-significant correlation (Spearman *ρ* = 0.22, *p* = 0.068) was found for all, both symptomatic and asymptomatic, carotid plaques. A significant difference in Spearman correlation coefficients between TBR and K^trans^ was found when grouped according to the symptomatic and asymptomatic carotid plaques (*p* = 0.033): a significant correlation (Spearman *ρ* = 0.59, *p* = 0.006) was found for symptomatic carotid plaques, not seen for asymptomatic plaques (Spearman *ρ* = 0.07, *p* = 0.625). Also, an inverse relationship was found between the time since the last neurological event and both parameters (Spearman *ρ* = −0.94 for TBR and Spearman *ρ* = −0.69 for K^trans^). These results point towards a complex interplay between inflammation and microvasculature in atherosclerotic plaques that is difficult to capture in clinical imaging.

The link between plaque microvasculature and the specific feature of intraplaque hemorrhage (IPH) has been investigated in symptomatic patients with moderate to severe carotid stenosis [45•]. The presence of IPH on MP-RAGE MR images was associated with a significant increase in K^trans^ of 28 % (*p* < 0.001) in the adventitial layer of the vessel wall as compared to arteries where IPH was absent (*p* < 0.001). A multivariate analysis adjusting for symptomatic status, degree of stenosis, and male sex showed that the increased K^trans^ in arteries with IPH remained significant (*p* = 0.018).

These studies show the potential of DCE-MRI as a tool to gain more insight in relation between plaque microvasculature and other features of vulnerable atherosclerotic lesions.

### Monitoring Longitudinal Changes in Plaque Microvasculature

DCE-MRI can be used to follow progression of atherosclerotic plaques, as illustrated by a preclinical study [31] of cholesterol-fed atherosclerotic rabbits. One group of rabbits was imaged 3 months after balloon denudation, immediately followed by euthanasia, and a second group at 3 and 6 months after balloon denudation. From 3 to 6 months after balloon denudation, an increase of 40 % in K^trans^ was found measured by DCE-MRI, suggesting that DCE-MRI can be used to investigate plaque microvasculature development.

### Differences Between Different Animal Groups and Human Subjects with a Different Cardiovascular Risk Profile

In a recent rabbit study [32], investigating the development of a microfluidic chip for potential future nanomedicines an increased AUC within the abdominal aorta for atherosclerotic animals as compared to control animals was reported. In another study of cholesterol-fed rabbits with induced plaque disruption [26], it was shown that ruptured plaques can be distinguished from stable plaques by spatial-temporal texture-based features of DCE-MRI. The effect of exposure to high particulate airborne matter on atherosclerosis was investigated in “Ground Zero” workers in New York City with high and low exposure to particulate matter using DCE-MRI [43]. Subjects with high exposure had a significantly higher AUC in the carotid artery (+41 %) as compared to subjects with low exposure (*p* = 0.016), indicating increased changes of the plaque microvasculature. These changes may range from increased leakage of contrast medium from the microvasculature, increased microvessel density, increased flow through the microvasculature, decreased reflux from the extracellular extravascular space to the microvasculature, or a combination. The authors of the study concluded that a high exposure to particulate matter may lead to increased plaque microvasculature, potentially indicating an increased risk for further development of atherosclerosis.

### Evaluation of Therapies

DCE-MRI enables the study of plaque microvasculature changes over time, making it useful in animal and patient drug effect studies. Changes in microvasculature may reflect changes in phenotype and/or vulnerability of the atherosclerotic plaque.

DCE-MRI has been employed in several preclinical cholesterol-fed balloon-injured atherosclerotic rabbit studies investigating potential anti-inflammatory treatments of atherosclerosis [33–35]. The effect of liposome-encapsulated prednisolone phosphate (L-PLP) on atherosclerosis was investigated using MR imaging before treatment, immediately after injection with L-PLP, and over time [33]. A reduction of the plaque AUC was found from pre-treatment to 2 days post-treatment, revealing early changes in microvascular permeability after treatment. In a further study, the anti-inflammatory effects of pioglitazone on atherosclerotic plaques were investigated [34]. DCE-MRI analysis showed a 22 % decrease in AUC for the treatment group (*p* < 0.01) over the study time period of 3 months, while no decrease in plaque enhancement was found for the control group. No changes in vessel wall area measurements were found during the study period for either animal group. A third study [35] evaluated the anti-inflammatory effects of a liver X receptor (LXR) agonist which induces reversal cholesterol transport, as compared to atorvastatin. The 3-month treatment with LXR did not lead to changes of the microvasculature, whereas treatment with atorvastatin caused a trend towards a decrease in microvasculature (*p* = 0.06). No differences in vessel wall area measurements were found. Combined, these studies have shown the potential of DCE-MRI to study changes of the plaque microvasculature in the evaluation of potential new therapies. A limitation of these studies, however, is that the rabbits did not exhibit plaque disruption with luminal thrombosis, the clinical endpoint of high risk plaques.

DCE-MRI has been used to study the effect of intensive lipid therapy over a period of 12 months [38] in patients with coronary artery disease or carotid disease and increased levels (≥120 mg/dl) of apolipoprotein B from the Carotid Plaque Composition study [70]. Results of the study show that 12-month therapy leads to a significant reduction of 21 % in K^trans^. This is consistent with the hypothesis that intensive lipid therapy results in a reduction of the extent and permeability of atherosclerotic plaque microvasculature. A study with 98 subjects with established cardiovascular disease [44••] selected from the AIM-HIGH trial [19] found an inverse association between v_p_ (plaque microvasculature fraction) and the duration of statin therapy. Statins are commonly used to lower lipid levels and also possess anti-inflammatory properties [71]. These results suggest that a relationship exists between duration of statin therapy and plaque microvasculature, which could reflect a decreased level of vascular inflammation.

The above studies on DCE-MRI of plaque microvasculature have measured differences between treatment groups or subjects with increased cardiovascular risk and shown that DCE-MRI can be employed effectively as an evaluation tool.

## Challenges and Future Perspectives in DCE-MRI of Atherosclerosis

To further advance DCE-MRI for wider use in clinical practice, uniform acquisition and analysis methods need to be agreed upon. Previous studies have shown that DCE-MRI-derived parameters are influenced by the contrast medium, vascular input function, and which pharmacokinetic model is used, making direct cross-study comparisons difficult. Use of a standard imaging and data analysis protocol is essential, therefore, for longitudinal studies of plaque microvasculature. A very important clinical precaution is use of a stable Gadolinium-based contrast medium [72] using low dosages to minimize the risks for nephrogenic systemic fibrosis and deposition of the contrast medium in the brain.

The recent introduction of interleaved acquisition methods [28••], providing both bright and black blood images, may be an important step toward the determination of an individualized vascular input function. In addition, 3D acquisition techniques [30•] may provide increased spatial accuracy as compared to currently employed 2D techniques, although at the expense of temporal resolution. Currently, these 3D acquisition techniques have only been explored in preclinical rabbit studies; their potential in clinical studies remains to be investigated.

All clinical DCE-MRI studies, to date, have been performed at 1.5 and 3.0 T. The potential of carotid MRI at 7.0 T has already been explored [73, 74], and results show a potential increase in signal-to-noise ratio (SNR) due to the increased field strength. However, the increase in SNR may be diminished by increased relaxivity of the contrast medium, which may also require longer scan times. The potentially increased SNR at 7.0 T would allow increased spatial and/or temporal resolution, but these studies also demonstrate that further technical developments are required to enable complete plaque characterization.

Associations between plaque microvasculature (measured using DCE-MRI) and plaque inflammation (measured by ^18^F-FDG uptake or macrophage content) remain an important area to be studied further since varying results have been reported to date. The reported association between and plaque microvasculature and intraplaque hemorrhage could be studied longitudinally. The recent introduction of hybrid PET-MRI systems provides excellent opportunities for further investigation of the relationships between these processes using a single imaging system. Recent research [75–79] has already shown the potential of hybrid PET-MR systems for the imaging of atherosclerosis. However, the additional value of DCE-MRI in PET/MR imaging is yet to be explored.

The predictive value of DCE-MRI for plaque progression or development of vulnerable plaque features is of great interest and remains to be determined; in addition, its predictive value for cerebrovascular ischemic events needs to be investigated in a prospective clinical trial.

Applications of DCE-MRI can be extended beyond the carotid artery to other (human) vascular territories, such as the microvasculature in the aortic wall of abdominal aortic aneurysms [80, 81]. These measurements were reproducible with a high technical success rate, and the Patlak model was the most suited pharmacokinetic model. Future studies are warranted to investigate the predictive potential of DCE-MRI derived parameters for abdominal aortic aneurysm rupture risk.

## Conclusion

Over the past decade, DCE-MRI has developed from a novel imaging tool to a useful non-invasive research tool used in animal and patient studies of plaque microvasculature. DCE-MRI has been used to investigate the relationship between plaque microvasculature and other plaque features such as inflammation and intraplaque hemorrhage, for assessing effectiveness of therapeutic interventions, and in the evaluation of plaque microvasculature changes over time and between groups with increased cardiovascular risks. Future studies could apply DCE-MRI to elucidate plaque development mechanisms, specifically the interplay between inflammation, increased microvasculature, and intraplaque hemorrhage. Also of great interest is the potential predictive value of plaque microvasculature DCE-MRI for plaque progression and future cerebrovascular ischemic events (such as stroke).

## Search Strategy

The studies discussed in the present review have been identified through a database search in MEDLINE in December 2015 using the following search terms: “carotid atherosclerosis”/“atherosclerosis”/“atherosclerotic plaque”/“atherosclerotic plaques”/“plaque” AND “human”/“rabbit” AND “DCE-MRI”/“dynamic contrast enhanced MRI”/“MRI”/“dynamic contrast enhanced magnetic resonance imaging”/“magnetic resonance imaging” AND “neovessels”/“neovascularization”/“neovasculature”/“vasa vasorum”/“microvasculature”/“inflammation”. Resulting abstracts and articles were screened and references checked for possible additional studies.
